# The Research Hospital at Cambridge

**Published:** 1908-12-12

**Authors:** 


### THE RESEARCH HOSPITAL AT CAMBRIDGE.

An appeal has recently been issued to the public and the profession on behalf of the research hospital at Cambridge. The history of this unique institution dates back some five or six years, when a small band of enthusiasts, of whom the most prominent was a well-known pathologist, collected some money by private appeals for medical research. It was decided to concentrate all the joint energy of the researchers upon one disease at a time, and "rheumatoid arthritis" appeared to be especially suitable, as being both common and ill-understood. About three years ago matters were sufficiently forward for the opening of a small hospital of five beds, in which subjects of this disease might be treated and systematically observed. The annual cost of keeping open this hospital is about £250, and it has now been decided to appeal for help to a wider public than has hitherto been reached. The appeal in question is signed by the Regius Professors of Oxford and Cambridge and by a number of those who stand highest in the ranks of the profession in London. On general grounds, therefore, we can have nothing but encouragement for this quite original method of setting about research. There is no reason why clinical investigation of this sort should not be carried on at Cambridge; nay rather, the large and well-equipped school offers such advantages along bacteriological lines that there is every reason why it should. At the same time it is not at first sight clear why Addenbrooke's hospital, to which the Regius Professor, Sir T. Clifford Allbutt, is himself physician, should not be made the scene of the work. The scheme has the official sanction and approval of the Professor. Why should it not be pursued in his wards? The difficulty which may be supposed to arise from his preoccupation with other business could easily be got over by the appointment as clinical assistants of those who are most interested in the matter. There is, again, the possibility that the governing body of Addenbrooke's might object; though there is no valid reason, so far as can be seen, why they should, if the merits of the scheme of research were carefully expounded. Be that as it may, the result of the appeal and of the experiment for which it is issued will be watched with much interest.

